# Gender differences in positive screen for depression and diagnosis among older adults in Chile

**DOI:** 10.1186/s12877-022-02751-y

**Published:** 2022-01-14

**Authors:** Ximena Moreno, Jean Gajardo, María José Monsalves

**Affiliations:** 1 Facultad de Psicología, Universidad San Sebastián, Lota 2465, Santiago, Chile; 2 Facultad de Ciencias para el Cuidado de la Salud, Universidad San Sebastián, Lota 2465, Santiago, Chile; 3 Departamento de terapia ocupacional y ciencia de la ocupación, Universidad de Chile, Independencia 1027, Santiago, Chile; 4 Facultad de Medicina y Ciencia, Universidad San Sebastián, Lota 2465, Santiago, Chile; 5 Area de Salud Pública, Instituto de Investigación en Ciencias Odontológicas, Facultad de Odontología, Universidad de Chile, Sergio Livingstone Pohlhammer 943, Santiago, Chile

**Keywords:** Depression, Ageing, Gender, Diagnosis, Mental health, Older adults

## Abstract

**Background:**

Different factors are associated with late life depression and diagnosis, including gender. It has also been reported that depression among older people is underdiagnosed. As a result, the mental health needs of this group are insufficiently met. The aim of this study was to explore gender differences in the factors associated with positive screens for depression and self-reported diagnosis among older adults in Chile.

**Methods:**

Data from 3786 older adults who participated in the Social Protection Survey in 2016 were analysed. PHQ-9 was used to identify screen-positive cases. Self-reported diagnosis of depression was used to determine the proportion of people with a screen-positive result who had received a diagnosis of depression. Logistic regression models were used to determine sociodemographic and health factors associated with depression and underdiagnosis in older men and women.

**Results:**

The prevalence of a screen-positive result was 20.91% (5.83% major depressive disorder) among men, and 36.38% (12.43% major depressive disorder) among women. 18.77% of men and 34.11% of women with a positive depression screening had received a diagnosis. More educated men were more likely to receive a diagnosis. Older age was associated with a lower probability of diagnosis among older women.

**Conclusions:**

Our results suggest that depressive disorders are undiagnosed in a high proportion of older adults in Chile. Gender is a relevant factor in the underdiagnosis of depression in this group. Further research is needed to understand the factors involved in these gaps, to improve detection and provide timely support and treatment.

## Background

Major depressive disorder (MDD) is the second most prevalent mental disorder (4.4%) [1], and the third leading global cause of years lived with disability YLD [2]. According to the World Health Organization [1], the prevalence of depression is higher in older adulthood, reaching above 7.5% among women and above 5.5% among men aged 55–74 years. Nevertheless, the prevalence of depression varies widely, depending on the regional and cultural context, and the assessment method used. Horackova et al. [3] reported 17–35% prevalence among older Europeans from different countries. Güzel & Kara [4] found a prevalence of 51.8% among older people in Turkey. In Latin America and the Caribbean, depressive symptoms ranged from 9.1% in Barbados to 36.8% in Chile [5]. Chile has the highest prevalence of depression in the region, and several initiatives have been implemented to address it [6, 7].

Among the several factors that are associated with depression, gender has been analyzed. A higher prevalence of depression is observed among women, across the lifespan [8–10]. However, the suicide rate is at least six times higher in older men with depression, as compared with women of the same age [11, 12]. Although the direction of the gender difference in depression in old age is consistent, the magnitude of this gap varies by cultural context [13]. The factors that explain these differences in old age are not well understood. Previous studies report conflicting results with respect to a greater vulnerability of women to social factors [14, 15]. There are factors that occur across the lifespan that may have an influence on depression in old age, but events that are more frequent at this age should also be considered, such as widowhood or health problems [9].

Late life depression has a negative impact on quality of life [16, 17] and is a risk factor for mortality [18, 19]. Nevertheless, there are important barriers to treatment, including underdiagnosis [20–22]. With respet to diagnosis of depression among older adults in Chile, a previous study reported that only 22.6% men and 42.3% women who screened positive for mild to severe depression had been diagnosed in 2009, according to their self-report [23]. To the best of our knowlege, gender differences in the factors associated with underdiagnosis of late life depression have not been analyzed previously. This study was aimed at exploring gender differences in the factors associated with positive screens for depression and self-reported diagnosis among older adults in Chile.

## Methods

### Sample

Secondary analyses of data from the Social Protection Survey (SPS) were carried out. The SPS is a fixed panel plus births longitudinal study that collects information about social security in Chile [24]. The SPS sample was recruited through multi-stage, stratified cluster sampling [24]. Since 2004, the sample has been nationally representative of people aged 18 or more years [25]. In 2015, a refreshment sample was recruited and weights were calculated, considering the projections of population to June 2015, made by the National Institute of Statistics [25]. Hence, the sample analyzed in this report was representative of the Chilean population in 2015. In the present study, data from the SPS sixth wave, collected in 2016, were analyzed, involving a total of 3786 people aged 60 years or older.

Since the present study performed secondary analyses of an anonymized and publicly available database, no ethical approval was required.

### Outcome measures

The 9-item Patient Health Questionnaire (PHQ-9) [26] was used as a depression screening. This instrument has performed well as a screening test for depression in primary care among adults [27] and older population [28] in Chile. A recent meta-analysis found that the PHQ-9 has high-sensitivity (0.88) and specificity (0.85) at a cutoff score of 10, compared with semi-structured diagnostic interviews to detect MDD [29]. Furthermore, the specificity increased with age. The PHQ-9 is a useful tool to detect the severity of depression, which is relevant for treatment decisions [26]. The PHQ-9 has nine items related to the presence of symptoms in the previous two weeks. The response categories and scores are: 0 (never), 1 (several days), 2 (more than half the days), and 3 (nearly every day). The cutoff scores to determine the severity of the symptoms are: 5–9 (mild) 10–14 (moderate), 15–19 (moderately severe), and 20–27 (severe) [30].

For analytic purposes, the previously validated cutoff score of 10 points or more [29] (Levis et al., 2019) was used to ascertain a screen-positive result for MDD. Analyses were also performed for any level of depression (score > 4).

Diagnosis of depression was self-reported. The question employed was: Have you been diagnosed with depression by a doctor?

### Covariates

Considering the relevant variables that may be associated with depression and depression diagnosis in old age [13], and those available from the SPS, specific sociodemographic and health variables were chosen.

Sociodemographic variables considered in the analyses were gender, age, educational attainment, living alone, and marital status. Age was categorized in three groups: 60–69, 70–79, and 80 or more years. Educational attainment was classified as less than 8 (incomplete primary education), 8–11 (complete primary education, but incomplete secondary education), and 12 or more years (complete secondary education or higher). Marital status included married, single, separated/divorced, and widowed. For each variable, the reference category was: man, 60–69 years of age, less than 8 years of education, not living alone, and married.

Health variables included were self-rated health, assessed through the question: Would you say your health is: excellent, very good, good, fair, poor, or very poor. The response categories were collapsed into good (good or better), and poor (fair or worse). Good was used as a reference. Disability was defined as needing help, or having difficulties performing any of the following basic activities of daily living: bathing, dressing, eating, and getting out of bed. The reference category was without disability. Self-report of the following chronic conditions was used: chronic obstructive pulmonary disease, diabetes, hypertension, cardiovascular disease, cancer, stroke, chronic kidney disease, and arthritis. The variable was categorized as none, 1–2, and 3 or more, using none as the reference.

### Statistical analyses

Due to the complex study design, weights were used in the analyses [24]. Chi square tests were employed to determine gender differences in sociodemographic and health characteristics of the sample. Prevalences and 95% confidence intervals of positive screen for MDD screen and for depressive symptoms (any level) were estimated, by gender.

Fully adjusted logistic regression models were estimated to determine the association between positive screen for MDD and sociodemographic and health variables in men and women. The covariates included in these models were: age, educational attainment, marital status, living alone, self-rated health, disability, and number of chronic diseases. The same procedure was used with a positive screen for depression of any level as the outcome. The factors associated with a diagnosis of depression among those participants with positive depression screening were explored with logistic regression models, stratified by gender, considering the same covariates as in the previous models.

All the analyses were performed with Stata 15.

## Results

As observed in Table 1, a higher proportion of men were married. Compared to men, women were more likely to be widowed, to live alone, and to have a poorer health status. Marital satus (2 cases, 0.1% of the sample), disability (9 cases, 0.2% of the sample), and self-reported diagnosis of depression (27 cases, 0.7%) had missing data. These cases were omitted in the logistic models.

**Table 1:** Sociodemographic and health characteristics of the sample

	Total (*N* = 3786)	Men (*N* = 1803)	Women (*N* = 1983)	*P*-value
Age				0.3
60–69	50.24	51.27	49.35	
70–79	32.43	32.85	32.06	
80+	17.33	15.87	18.59	
Education				0.09
Less than 8	38.22	36.60	39.62	
8–11	27.68	26.94	28.31	
12 or more	34.11	36.46	32.07	
Marital status				< 0.001
Married	55.56	70.13	42.89	
Single	11.30	8.54	13.70	
Separated/Divorced	9.34	8.48	10.10	
Widowed	23.80	12.85	33.31	
Lving alone				0.001
Yes	16.51	13.75	18.91	
Self-rated health				< 0.001
Good	35.91	40.36	32.06	
Poor	64.09	59.64	67.94	
Disability				< 0.001
Yes	23.06	17.72	27.70	
Chronic conditions				< 0.001
0	27.82	34.44	22.07	
1–2	54.85	53.94	55.64	
3 or more	17.33	11.62	22.29	

The prevalence of a positive screen for MDD was 9.37% (95% CI 8.25–10.62) (Table 2). The prevalence was more than twice as high in women (12.43, 95% CI 10.72–14.38) than in men (5.83, 95% CI 4.58–7.40). Positive screen for mild depressive disorder was also more prevalent among women (23.95, 95% CI 21.71–26.33), as compared with men (15.08, 95% CI 13.19–17.19). The same gender difference was observed when considering all levels of severity of depression, with women reaching 36.38% (95% CI 33.75–39.09), and men, 20.91% (95% CI 18.66–23.35).

**Table 2:** Prevalence of positive screen for depressive disorders among Chilean older men and women, and proportion who had received a diagnosis of depression

	Total	95% CI	Men	95% CI	Women	95% CI
Major depressive disorder (≥10)	9.37	8.25–10.62	5.83	4.58–7.40	12.43	10.72–14.38
% Diagnosed	39.56	33.26–46.23	32.11	20.99–45.72	42.70	35.27–50.5
Mild depressive disorder (5–9)	19.83	18.32–21.43	15.08	13.19–17.19	23.95	21.71–26.33
% Diagnosed	24.08	20.65–27.88	13.54	9.66–18.65	29.78	25.12–34.90
Any depressive disorder (≥5)	29.19	27.40–31.05	20.91	18.66–23.35	36.38	33.75–39.09
% Diagnosed	28.99	25.81–32.39	18.77	14.26–24.29	34.11	30.04–38.42

A lower proportion of men (18.77, 95% CI 14.26–24.29), among those who screened positive for any level of severity of depression, had received a medical diagnosis of depression, as compared with women (34.11, 95% CI 30.04–38.42). Among men with a positive screening for MDD, 67.89% were undiagnosed, whereas 57.30% women were in the same case.

Positive screen for MDD was associated with health status in both men and women (Table 3). People with a negative self-rating of health and disability were more likely to screen positive for depression, in both sexes. Additionally, having three or more chronic diseases was associated with positive screen for MDD among men (OR = 3.79; 95% CI 1.67–8.59). The number of chronic conditions was also associated with depression of any level of severity in men. Additionally, more educated men were less likely to have a positive screen for depression of any level of severity (OR = 0.67; 95% CI 0.45–0.99). Men (OR 0.70; 95% CI 0.49–0.99) and women (OR = 0.59; 95% CI 0.44–0.78) aged 70–79 years were less likely to screen positive for any level of depression, as compared with the youngest group, and having three or more chronic conditions was associated with a positive screen for depressive symptoms among both genders (men: OR = 2.73; 95% 1.70–4.37; women: OR = 2.15; 95% CI 1.42–3.26).

**Table 3:** Adjusted odds ratios and 95% confidence intervals of positive screen for depression among Chilean older men and women

	Major depressive disorder				Depressive disorder of any level of severity			
	Men		Women		Men		Women	
	OR	95% CI	OR	95% CI	OR	95% CI	OR	95% CI
Age (60–69)								
70–79	1.00	0.55–1.82	0.67	0.44–1.01	0.70	0.49–0.99	0.59	0.44–0.78
80+	1.18	0.60–2.34	0.99	0.54–1.83	1.13	0.71–1.81	0.83	0.55–1.24
Years of education (< 8)								
8–11	1.58	0.88–2.81	0.94	0.61–1.46	1.21	0.86–1.71	0.93	0.69–1.26
12+	1.58	0.84–2.95	1.19	0.77–1.84	0.67	0.45–0.99	0.81	0.60–1.10
Marital status (married)								
Single	0.93	0.40–2.16	1.33	0.72–2.46	1.03	0.59–1.81	0.93	0.62–1.40
Divorced	0.52	0.17–1.55	1.25	0.67–2.32	0.89	0.4–1.98	1.36	0.89–2.08
Widowed	0.53	0.22–1.27	1.37	0.84–2.23	1.22	0.74–1.99	1.22	0.88–1.70
Living alone	2.13	1.00–4.52	1.17	0.74–1.84	1.21	0.72–2.05	1.26	0.91–1.74
Negative self-rated health	5.45	2.37–12.52	10.02	5.08–19.77	3.06	2.04–4.59	4.25	3.03–5.96
Disability	2.11	1.19–3.76	2.14	1.45–3.15	2.29	1.63–3.21	2.23	1.68–2.98
Chronic conditions (none)								
1–2	1.40	0.70–2.8	1.63	0.86–3.08	1.55	1.05–2.27	1.42	0.98–2.06
3+	3.79	1.67–8.59	1.69	0.87–3.29	2.73	1.70–4.37	2.15	1.42–3.26

With respect to diagnosis, the highest level of education was associated with a higher odds of depression diagnosis among older men with a positive screen for MDD (Table 4). For depression of any level of severity, having 12 or more years of education was associated with a medical diagnosis in men (OR = 3.32; 95% CI 1.52–7.23), whereas widowhood decreased the likelihood of having received a diagnosis (OR = 0.32; 95% 0.11–0.93). Among those women with a positive screen for MDD, being 80 years or older decreased the likelihood of receiving a diagnosis (OR = 0.08; 95% CI 0.03–0.23). The same occurred among women who screened positive for any level of depression (OR = 0.17; 95% CI 0.09–0.33), and a negative self-rating of health increased the likelihood of medical diagnosis of depression among them (OR = 2.09; 95% CI 1.10–3.97).

**Table 4:** Adjusted odds ratios and 95% confidence intervals of self-reported diagnosis of depression among Chilean older men and women with positive depression screening

	Major depressive disorder				Depressive disorder of any level of severity			
	Men		Women		Men		Women	
	OR	95% CI	OR	95% CI	OR	95% CI	OR	95% CI
Age (60–69)								
70–79	0.76	0.17–3.44	0.86	0.39–1.91	0.99	0.44–2.21	0.77	0.49–1.21
80+	0.77	0.14–4.38	0.08	0.03–0.23	0.58	0.25–1.35	0.17	0.09–0.33
Years of education (< 8)								
8–11	5.56	1.06–29.22	0.59	0.26–1.33	1.60	0.77–3.33	0.96	0.60–1.51
12+	13.67	2.42–77.09	1.38	0.57–3.33	3.32	1.52–7.23	1.52	0.92–2.50
Marital status (married)								
Single	2.29	0.39–13.30	0.77	0.22–2.61	0.93	0.25–3.42	0.88	0.43–1.79
Divorced	3.42	0.35–32.95	2.11	0.65–6.99	1.31	0.30–5.67	1.0	0.54–1.86
Widowed	0.84	0.11–6.68	2.16	0.89–5.21	0.32	0.11–0.93	1.40	0.83–2.36
Living alone	1.87	0.33–10.6	2.46	0.99–6.02	1.70	0.50–5.71	1.27	0.75–2.15
Negative self-rated health	1.59	0.19–13.20	2.21	0.64–7.65	2.04	0.82–5.07	2.09	1.10–3.97
Disability	0.98	0.29–3.31	1.64	0.75–3.63	1.05	0.51–2.15	1.04	0.68–1.58
Chronic conditions (none)								
1–2	1.93	0.36–10.53	0.67	0.20–2.22	1.52	0.63–3.69	0.62	0.32–1.18
3+	3.95	0.58–26.80	1.03	0.28–3.83	2.29	0.80–6.60	1.32	0.67–2.60

## Discussion

Late life depression is a major public health concern, due to its significant impact in quality of life, health trajectories and survival [16, 19, 31]. This study sought to examine the gender differences in positive screens and self-reported diagnosis of depression in older adults in Chile, as a contribution to understand the factors involved in underdiagnosis.

A positive screening for depression was associated with health problems in men and women. It could be hypothesized that older people with more health problems are more likely to be in contact with health services, but a negative self-rating of health, disability, or a higher number of chronic diseases did not increase the odds of having received a diagnosis of depression. For any level of depression, we found that less than one fifth of older men and only one third of older women who screened positive, self- reported a diagnosis of depression. Considering those who screened positive for MDD, less than half had been diagnosed, according to their self-report. These results support the existence of a substantial gap between the prevalence of depressive disorders and their diagnosis and treatmentt, among the Chilean older population.

Our results contribute to the previous information about depressive disorders among older adults in Chile. Aravena et al. [23] found a prevalence of positive screen for MDD of 6.9% in this group in 2009, assessed with the 15-item Geriatric Depression Scale (GDS-15). Our study found a higher prevalence (9.4%), which could be attributed to the use of a different screening test. However, a previous study found that the GDS-15 performed similarly to the PHQ-9 among older adults without neurocognitive disorders [32]. Furthermore, another survey in Chile found a similar prevalence of depressive disorders among older people in 2019, using both the PHQ-9 and GDS-15 (UC & Caja Los Andes, 2020). Accordingly, the prevalence of MDD was 10.2%, suggesting a progressive increase in MDD among Chilean older people during the last decade.

With respect to sociodemographic variables, Aravena et al. [23] reported that female gender, having a lower level of education, and living alone were associated with depression, whereas older age decreased the odds of depression. We observed that gender alone was associated with a positive screen for MDD (data not shown). Higher levels of education, and being between the ages of 70–79 years-old, decreased the odds of a positive screen for depression of any level (data not shown). However, the gender-stratified analyses showed that a higher educational attainment decreased the odds of a positive screen for depression among men, but not among women. Considering that a recent systematic review of factors associated with depression in older adults found heterogeneous results for men and women, with respect to education [33], further research is needed to explore this association.

Men with a positive screen for any level of depression were less likely to self-report a medical diagnosis, as previously found by Aravena et al. [23]. As these authors had reported, we observed associations between age, educational attainment, and the odds of diagnosis, but we found that older women were less likely to self-report a depression diagnosis, and that educational attainment increased this likelihood only among men.

The important treatment gap for depression in Chile was identified in the early 2000’s, showing inequities in the distribution of mental health provision [34]. In 2005, the Regime of Explicit Health Guarantees (AUGE) was introduced, aimed at providing access, quality, opportunity, and financial protection for prioritised health conditions in Chile, including depression [35]. As a result, once a person is diagnosed with depression, access to treatment within a specific period of time is guaranteed by law [36]. An analysis by Araya et al. [35] suggested that access to health-care among people who screened positive for MDD had increased in Chile between 2003 and 2010. According to the authors, people with depression aged 60 years and older reported a more frequent acces to health-care, reaching 71.5% in 2010 [35]. We observed a wider gap, with only 39.6% self-reported diagnosis among people with a positive screen for MDD. Aravena et al. [23] reported that 47% of people who screened positive for moderate to severe depression in 2009, had been diagnosed. Hence, the gap between a positive screen for depression and diagnosis among older Chilean people should be addressed by mental health programs. In Chile, there is a unique protocol to monitor and treat depression in people aged 15 and older, but no tailored interventions for older adults have been developed in the context of the national mental health programs. Recently, an increasing awareness of the urgency of mental health needs of older adults in Chile has been observed. Several initiatives to disseminate information about this topic have been observed, including a proposal to address mental health problems and a guide to prevent suicide among older adults in Chile [37, 38]. Also, the National Service for Older Adults (Servicio Nacional de Adulto Mayor, SENAMA) has formed an intersectoral committee on mental health in older adults, providing a series of recommendations to implement a mental health strategy for older adults in Chile [39]. However, these initiatives are still a first approach, and their impact on diagnosis and access to treatment is yet to be determined. More research is needed to better understand the factors involved in the underdiagnosis of depression in old age and the gender differences in prevalence, access to diagnosis and treatment, and suicide risk among older adults in Chile. This information would be a contribution to the development of protocols, the allocation of new resources, and the design of relevant and effective tools to train mental health providers.

This study has some limitations. Although the PHQ-9 has shown good sensitivity and sensibility as a screening test for MDD [29], a meta-analysis found that when this instrument is used in population surveys, it overestimates the prevalence of MDD by an average of 2.5 times [40]. A positive screen of MDD with the PHQ-9 should be confirmed with a clinical interview. Nevertheless, in a clinical setting, a positive screen should always be followed-up to determine the presence of MDD, other depressive disorders or mental health problems that require treatment or monitoring [41]. Also, recall bias cannot be ruled out for the information about a medical diagnosis of depression, which was self-reported. However, as mentioned above, people who receive a diagnosis of depression in Chile have access to treatment by a specific time [36]. The subsequent monitoring of these guarantees could reduce the likelihood of this bias. It is also possible that some people may have preferred not to report a diagnosis of depression, due to the existing negative stereotypes about mental health problems in Latin America [42], which could have led to an overestimation of the gap between a positive screen for MDD and diagnosis. Our results should be interpreted taking these limitations into account.

## Conclusions

Health status was associated with a positive screen for depression in men and women. Our results suggest that depressive disorders are underdiagnosed in a high proportion of older adults in Chile. Gender was a relevant factor in the underdiagnosis of depression in this group, with a lower proportion of diagnosis among men who screened positive for depression. In our study, less educated men and women aged 80 years or more, were less likely to receive a diagnosis. Further research is needed to understand the factors involved in these gaps, to improve detection and provide timely support and treatment.

## Data Availability

The datasets generated and/or analysed during the current study are available in the database repository of the Social Protection Undersecretary from the Ministry of Labor and Social Security: https://www.previsionsocial.gob.cl/sps/biblioteca/encuesta-de-proteccion-social/bases-de-datos-eps/
